# Individualized ranibizumab therapy strategies in year 3 after as-needed treatment for polypoidal choroidal vasculopathy

**DOI:** 10.1186/s12886-015-0026-y

**Published:** 2015-04-10

**Authors:** Taiichi Hikichi

**Affiliations:** Ohtsuka Eye Hospital, Kita-16 Nishi-4, Kita-ku, Sapporo, 001-0016 Japan

**Keywords:** Individualized therapy, Polypoidal choroidal vasculopathy, Ranibizumab

## Abstract

**Background:**

To investigate the third-year results of ranibizumab monotherapy for polypoidal choroidal vasculopathy (PCV) in individualized treatment regimens based on the outcomes during 2 years.

**Methods:**

One hundred seventy-two consecutive eyes of 163 prospective treatment-naïve patients with PCV were treated with three monthly intravitreal ranibizumab injections followed by as-needed reinjections and completed a 2-year follow-up. Treatment regimens during the third year were selected individually based on their outcomes from the following treatment regimens: as-needed injections based on quarterly examinations, as-needed injections based on monthly examinations, a monthly ranibizumab injection schedule, and the treat-and-extend schedule. Visual acuity (VA) and foveal thickness at the end of the third year and the prevalence of discontinuous follow-up examinations during the third year were evaluated.

**Results:**

Of 163 patients, 35 (21%) patients were excluded; nine patients had discontinuous follow-up examinations during the third year. In 128 eyes of 128 patients studied during the third year, the significant improvements in VA and foveal thickness 2 years after the first injection compared to baseline were maintained at the end of the third year. Six (18%, 6/34) patients treated with as-needed injections based on quarterly examinations had discontinuous follow-up examinations, the prevalence of which differed significantly (*P* = 0.025) from the other groups.

**Conclusions:**

The individualized treatment strategies in the third year based on each patient’s outcomes during 2 years maintained the improved VA and avoided discontinuation of follow-up during the third year.

## Background

The Seven-Year Observational Update of Macular Degeneration Patients Post-MARINA [1] (the Minimally Classic/Occult Trial of the Anti-VEGF [vascular endothelial growth factor] Antibody Ranibizumab in the Treatment of Neovascular Age-Related Macular Degeneration [AMD])/ANCHOR [2] (the Treatment of Predominantly Classic Choroidal Neovascularization in Age-related Macular Degeneration) and HORIZON [3] (the Open-Label Extension Trial of Ranibizumab for Choroidal Neovascularization Secondary to Age-Related Macular Degeneration) trials [4] was conducted to assess the long-term visual acuity (VA) outcomes and disease status 7 to 8 years after initiation of intensive ranibizumab (Lucentis, Genentech Inc., South San Francisco, CA) therapy and evaluate the risk of late visual decline over years 4 to 8 since leaving the HORIZON study [3]. At a mean 7.3 years after initiation of intensive ranibizumab treatment, about one third of patients had a VA of 20/70 or better Snellen equivalent, and almost one quarter had good vision (20/40 or better). However, a third had poor vision (20/200 or worse). In half of patients, the fellow non-study eyes also had wet AMD, and 6% of patients were legally blind, with 20/200 vision or worse bilaterally. The results suggested that to maintain the VA gain in patients with neovascular AMD treated with ranibizumab, patients may have to be followed more frequently than quarterly or treated more frequently. Recent trials [3-7] have reported that as-needed ranibizumab injections based on monthly examinations could be effective if patients strictly adhered to the prespecified objective treatment criteria, which requires intensive long-term follow-up.

A previous study reported that monthly intravitreal injections of ranibizumab for 3 months followed by an as-needed reinjection schedule resulted in continued VA improvement that was maintained throughout 2 years of follow-up in eyes with polypoidal choroidal vasculopathy (PCV) [8]. Although the mean ± standard deviation (SD) total number of injections during 2 years was 5.6 ± 1.9 and the patients visited our clinic monthly according to an as-needed reinjection schedule based on monthly examinations, the numbers of reinjections and the changes in VA varied among patients. Because PCV is a chronic disease and patients must be followed over the long term [9,10], a strict treatment regimen is essential to maintain favorable VA outcomes with ranibizumab therapy and quality of vision and life [11-13]. However, it is difficult to continue strict treatment regimens over the long term. The treatment burden of aggressive as-needed injections includes the injections themselves, monthly visits (required for the optimal efficacy of as-needed injection regimens), ancillary examinations to evaluate the retreatment efficacy, and interpretation of the results based on the retreatment criteria. Further, since the degree of patient satisfaction with the results of ranibizumab treatment and the status of the fellow eye vary, adherence to and motivation to continue ranibizumab treatment likely also vary among patients. The risk of a marked decrease in VA has been reported during discontinuation of follow-up examinations, indicating that regular examinations are warranted and that the major reasons for discontinuation were sustained low VA and lack of apparent treatment response [14].

Thus, at the end of the second year of an as-needed reinjection schedule based on monthly examinations, to avoid losing patients to follow-up, continue treatment, and maintain the VA gains achieved during the 2-year treatment, the treatment schedule for the third year was changed from an as-needed reinjection schedule based on monthly examinations of all patients to treatment schedules based on the clinical course during the 2-year follow-up period in each patient. The current study reports the results after 3 years of ranibizumab therapy, i.e., three monthly injections followed by as-needed reinjections based on monthly examinations for 2 years and an individualized treatment regimen for the third year based on the clinical course during the 2-year follow-up period.

## Methods

One hundred seventy-two consecutive eyes of 163 prospective treatment-naïve Japanese patients with symptomatic PCV who received monthly intravitreal injections of 0.5 mg of ranibizumab for 3 months followed by a reinjection schedule based on need were followed for 2 years.

PCV was defined as the presence of one or multiple focal areas of hyperfluorescence arising from the choroidal circulation within the first 6 minutes after injection of indocyanine green with or without an associated branching vascular network. Polypoidal lesions are solitary (arbitrarily defined as one or two polyps) or multiple, i.e., arranged in a ring or cluster [15-17]. There were no exclusion criteria regarding the baseline VA or lesion size. Most eyes in the current study were in our previous study [8] that reported the 2-year outcomes of ranibizumab monotherapy for PCV. Reinjections were administered during the third year if any of the following occurred [7,8]: visual loss determined using a decimal VA chart, with fluid at the macula seen on optical coherence tomography (OCT) images; any qualitative changes seen on the OCT images that suggested recurrent fluid in the macula including enlargement of a pigment epithelial detachment; new macular hemorrhages; or persistent fluid in the OCT images 1 month after the previous injection. All criteria were based on comparisons with the previous examination. Since an as-needed injection protocol was much tighter than that used previously and is often referred to as “zero tolerance”, [18] any fluid seen on OCT images was added to the usual retreatment criteria. When a reinjection was required, intravitreal ranibizumab was administered on the same day as the examination.

At the end of the second year of ranibizumab therapy, one of the following four treatment schedules was adopted during the third year for each patient after they provided informed consent: 1) as-needed injections based on quarterly examinations were administered if two or fewer reinjections had been administered during the second year, the treated eye was the nondominant eye with 0.1 decimal VA (equivalent to 20/200 Snellen VA) or worse, and the VA of the dominant fellow eye was 0.7 decimal VA (equivalent to about 20/30 Snellen VA) or better; 2) as-needed injections based on monthly examinations were continued even if two or fewer reinjections had been administered during the second year and the patients did not meet the previous criterion; 3) if five or more reinjections had been administered during the second year, a monthly ranibizumab injection schedule was adopted that included the dominant eye or the eye with a VA decrease of 0.3 logarithm of the minimum angle of resolution (logMAR) unit converted from the decimal VA or more from the baseline logMAR VA during the 2-year follow-up period of as-needed treatment based on monthly examinations; and 4) the treat-and-extend schedule [19] was adopted if three or four reinjections had been administered during the second year or the patients did not meet the third criterion even if five or more reinjections had been administered during the second year.

The institutional review board of Ohtsuka Eye Hospital approved the treatment strategy. The current research followed the tenets of the Declaration of Helsinki. All patients provided informed consent after explanation of the study protocol. Since aflibercept (Eylea, Regeneron, Tarrytown, NY, and Bayer, Berlin, Germany) and bevacizumab (Avastin, Genentech, Inc.) had not yet been approved at the end of the second year of this study in Japan, switching the anti-VEGF agents could not be considered as a treatment strategy for neovascular AMD.

In cases with bilateral disease, the eye treated first with ranibizumab was included in the current study. The VA was measured with the Landolt ring chart. Digital simultaneous fluorescein angiography and indocyanine green angiography images were obtained by scanning laser ophthalmoscopy (Heidelberg Retina Angiograph II, Heidelberg Engineering Inc., Dossenheim, Germany) throughout the study. The foveal thickness was based on the average foveal thickness on the vertical and horizontal scans of OCT (OCT 3000, Zeiss Humphrey Instruments, Dublin, CA or Spectralis, Heidelberg Engineering Inc.). The VA was measured and OCT was performed at every visit during the 3-year follow-up period.

The logMAR unit calculated from the decimal VA was used to analyze the VA. Statistical analysis was performed using SPSS 11.5.1 for Windows software package (SPSS Inc., Chicago, IL). Multiple comparisons were performed by one-way analysis of variance with the Scheffe’s F test. *P* < 0.05 was considered significant.

## Results

Of 163 prospective treatment-naïve Japanese patients who received ranibizumab therapy for 2 years, 18 patients chose to return to the clinics that referred them to our institute for treatment of PCV at the end of the 2-year treatment period. Eight patients were excluded from the current study, because even though the patients met the criteria of the monthly treatment schedule, they could not return for monthly visits during the third year because of proximity to the clinic, absence of available ophthalmic clinicians, or the treatment schedule for as-needed injections based on quarterly examinations was selected for the third-year treatment. Nine patients had discontinuous follow-up examinations during the third year. Thus, 128 (79%, 128/163) eyes of 128 patients (mean ± SD age, 73 ± 8 years; range, 47–89 years) were studied in the analysis of the clinical outcomes during the third year.

During the third year, 28 eyes received as-needed injections based on quarterly examinations, 61 eyes received as-needed injections based on monthly examinations, 18 eyes received monthly ranibizumab injections, and 21 eyes had a treat-and-extend schedule. Table 1 shows the mean age and the number of ranibizumab injections in each treatment group. Since the study eyes were divided into four groups depending on the number of injections during the second year, the numbers of injections during the second and third years differed significantly (*P* = 0.01) between every two groups.

**Table 1:** **The mean (± standard deviation) age and number of ranibizumab injections in each treatment schedule group**

**Treatment schedule**		**No. injections**		
**During the third year**	**Age**	**During the first year including three monthly injections**	**During the second year**	**During the third year**
All eyes (n = 128)	73 (8)	4.2 (1.3)	2.2 (1.9)	3.9 (4.1)
As-needed- injections determined by quarterly examination schedule				
(n = 28)	73 (8)	4.3 (1.6)	0.6 (0.9)	1.0 (0.9)
As-needed injections determined by monthly examinations schedule				
(n = 61)	71 (8)	4.0 (1.2)	1.5 (0.9)	1.8 (1.1)
Monthly injection schedule (n = 18)	75 (8)	4.3 (1.8)	5.5 (0.8)	11.4 (0.7)
Treat-and-extend schedule (n = 21)	78 (7)	4.6 (1.0)	3.8 (0.6)	6.4 (2.4)

**Figure 1:**
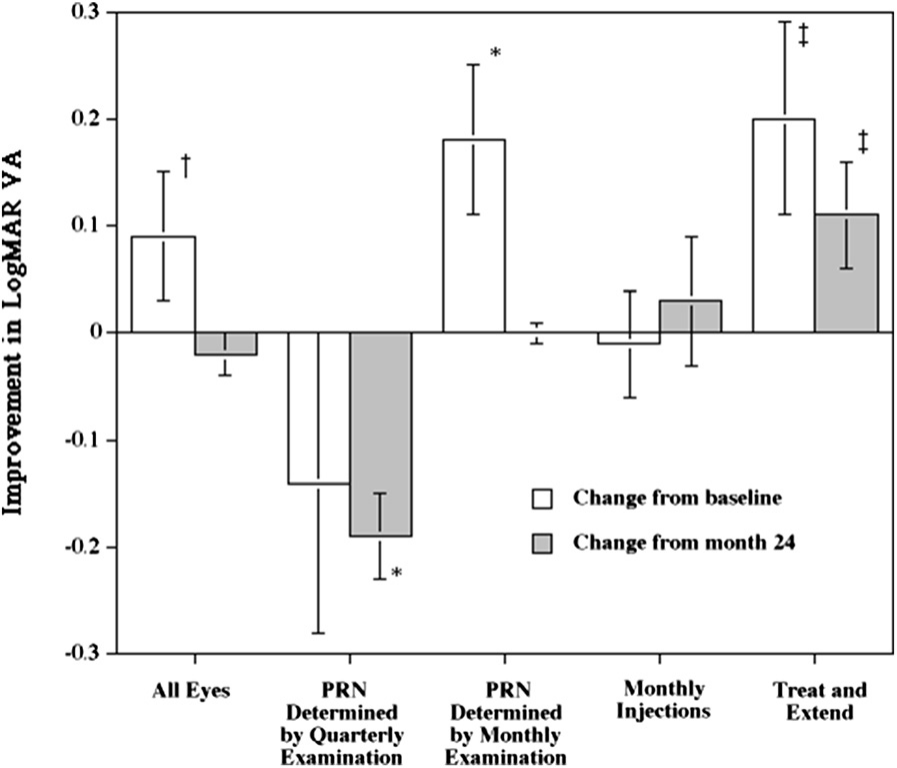
**Improvement in the logarithm of the minimum angle of resolution (logMAR) visual acuity (VA) at the end of the third year in each treatment schedule.** The white and shaded bars indicate the changes from baseline and the 2-year values, respectively. The positive values on the Y-axis indicate VA improvements at the end of the third year compared to baseline or 2 years. PRN = pro re nata. *P = 0.01; †*P* = 0.04; ‡*P* = 0.02.

Table 2 shows the mean (± SD) logMAR VA at various times after the first injection. In 128 eyes, the VAs at 1, 2, and 3 years after the first injection significantly (*P* = 0.01, *P* = 0.01, and *P* = 0.04, respectively) improved compared to the baseline VA. Although the VA decreased significantly (*P* = 0.01) during the second year, the VA at the end of the second year was maintained at the end of the third year (Figure 1). In eyes treated with as-needed injections based on quarterly examinations during the third year, the baseline VA was the worst among the four groups, but the baseline VA was maintained at the end of the third year. However, the VA 3 years after the first injection decreased significantly (*P* = 0.01) compared to the VA 2 years after the first injection. In eyes treated with as-needed injections based on monthly examinations during the third year, the VAs at 1, 2, and 3 years after the first injection improved significantly (*P* = 0.01, for all comparisons) compared with the baseline VA. Although the VA decreased significantly (*P* = 0.01) during the second year, no difference was found in the VA between 2 and 3 years after the first injection. In eyes receiving monthly injections, the baseline VA was relatively good, but the VA 1 year after the first injection improved significantly (*P* = 0.03) compared with the baseline VA. Although the VA decreased significantly (*P* = 0.01) during the second year, no difference was found in the VA between years 2 and 3 after the first injection and the monthly injection maintained the VA during the third year (Figure 1). In eyes on the treat-and-extend schedule, the VA at 1 year after the first injection improved significantly (*P* = 0.03) compared with the baseline VA. The treat-and-extend treatment during the third year significantly (*P* = 0.01) improved the VA compared to the VA after the second year, which was significantly decreased (*P* = 0.02) during the second year with as-needed injections based on monthly examinations.

**Table 2:** **The mean (± standard deviation) visual acuity of the logarithm of the minimum angle of resolution at various points after the first injection in each treatment schedule group**

**Treatment schedule**				
**During the third year**	**Baseline**	**1 year**	**2 years**	**3 years**
All eyes (n = 128)	0.61 (0.51)	0.43 (0.43)	0.50 (0.42)	0.52 (0.47)
As-needed injections determined by quarterly examination schedule				
(n = 28)	0.98 (0.58)	0.89 (0.46)	0.93 (0.45)	1.12 (0.49)
As-needed injections determined by monthly examination schedule				
(n = 61)	0.48 (0.49)	0.23 (0.30)	0.30 (0.32)	0.30 (0.30)
Monthly injections schedule (n = 18)	0.34 (0.08)	0.21 (0.18)	0.38 (0.17)	0.35 (0.12)
Treat-and-extend schedule (n = 21)	0.72 (0.38)	0.55 (0.27)	0.63 (0.26)	0.52 (0.22)

**Figure 2:**
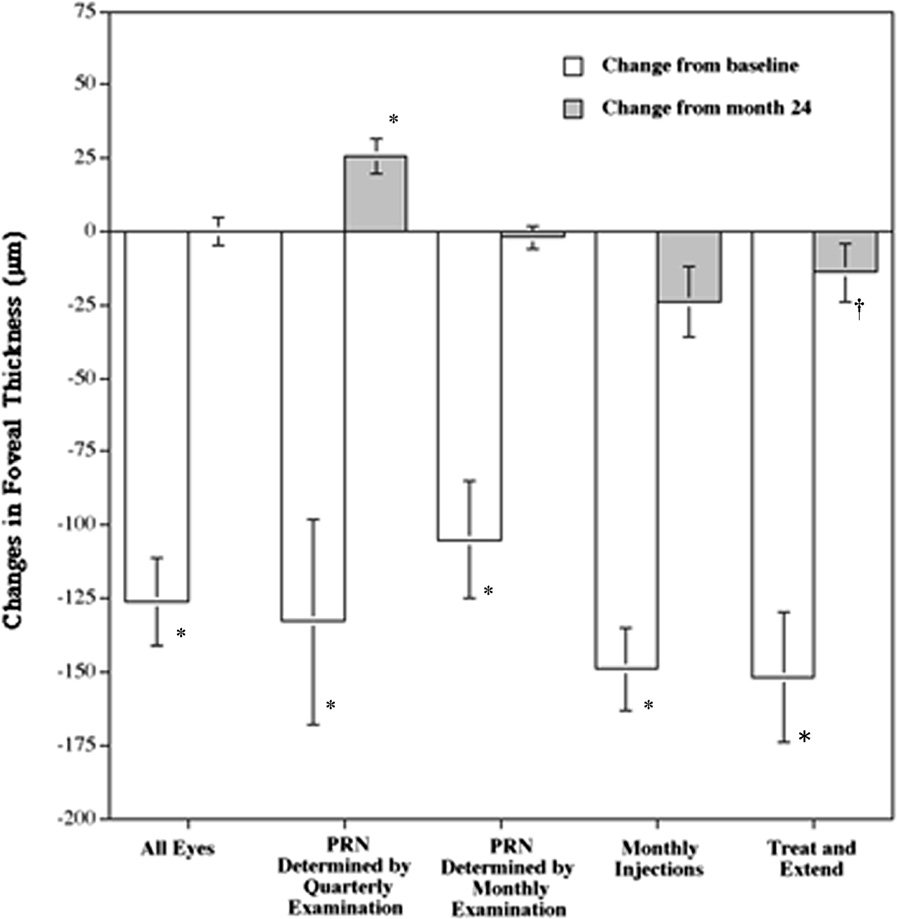
**Changes in the foveal thickness at the end of the third year in each treatment schedule.** The white and shaded bars indicate changes from the baseline and the 2-year values, respectively. PRN = pro re nata. *P = 0.01; †*P* = 0.03.

Table 3 shows the mean (± SD) foveal thicknesses at various times after the primary injection. In the 128 eyes and all four treatment groups during the third year, the foveal thicknesses at 1, 2, and 3 years after the first injection were significantly (*P* = 0.01, for all comparisons) thinner compared to baseline. No apparent differences were seen in the foveal thicknesses between years 2 and 3 in each treatment group, except that the mean foveal thickness at 3 years was significantly thicker compared to that at 2 years in the group treated with as-needed injections based on quarterly examinations and thinner compared to that at 2 years in the group treated with monthly injections (*P* = 0.01 and *P* = 0.04, respectively) (Figure 2).

**Table 3:** **The mean (± standard deviation) foveal thickness (μm) at various points after the first injection in each treatment schedule group**

**Treatment schedule**				
**During the third year**	**Baseline**	**1 year**	**2 years**	**3 years**
All eyes (n = 128)	338 (127)	206 (69)	212 (62)	212 (55)
As-needed injections determined by quarterly examination schedule				
(n = 28)	349 (154)	206 (60)	190 (39)	216 (41)
As-needed injections determined by monthly examination schedule				
(n = 61)	303 (120)	201 (73)	200 (65)	198 (53)
Monthly injection schedule (n = 18)	380 (122)	214 (72)	255 (49)	231 (48)
Treat-and-extend schedule (n = 21)	377 (73)	213 (70)	239 (68)	225 (73)

At the beginning of the third year, 137 patients participated in this study, but nine patients discontinued follow-up examinations during the third year. Thus, 93% (128/137) of patients continued the third-year examinations. Of the nine eyes, six (18%, 6/34) eyes were treated with as-needed injections based on quarterly examinations, two (3%, 2/63) eyes were treated with as-needed injections based on monthly examinations, and one (5%, 1/22) eye was treated with the treat-and-extend schedule. The numbers of the patients who had discontinuous follow-up examinations differed significantly (*P* = 0.04) among the groups.

No endophthalmitis, uveitis, lens damage, or prolonged intraocular pressure elevations occurred. Ten eyes underwent cataract surgery during the third year. In three eyes, the presence of minute amounts of vitreous hemorrhage was thought to be associated with the injection site at the uvea but spontaneously resolved within a few days without medication.

## Discussion

In this study, the treatment schedule during the third year in each patient was based on the 2-year results with as-needed injections, monthly examinations, and the VA of the fellow eye. The improved VA in the 128 study eyes at the end of the second year compared to the baseline VA was maintained at the end of the third year. Ninety-three percent of the patients who participated in this study could have continued the follow-up examinations during the third year.

Since PCV is a chronic disorder [9,10], a choroidal branching vascular network remains after anti-VEGF therapy even when combined with photodynamic therapy [8,11-13,20], which can result in recurrent polypoidal lesions, and clinical examinations are necessary for the patients’ lifetimes. Because the fundus findings and natural histories vary in eyes with PCV [9,10], the numbers of required injections during the second year also varied among eyes with PCV (Table 1). Furthermore, the VAs at the end of the second year varied among the treated eyes and the VAs of the fellow eyes probably differed in each patient. Retina specialists who treat patients with AMD must maintain the patients’ activities of daily living (ADL) while considering the treatment outcomes and burden and medical expenses each patient pays [21]. Thus, at the end of 2 years of ranibizumab therapy, the treatment regimen in the third year in eyes with PCV was changed from as-needed injections based on monthly examinations to individualized treatment strategies according to each patient’s clinical course during the 2-year follow-up period.

In eyes treated with as-needed injections based on monthly examinations, the monthly injection schedule, and the treat-and-extend schedule in the third year, VAs decreased significantly during the second year, but individualized treatment strategies during the third year could avoid the decreased VA. In eyes treated with the treat-and-extend schedule, VA improved significantly during the third year. On the contrary, in eyes treated with as-needed injections based on quarterly examinations, VA decreased significantly during the third year. The outcomes of the four treatment strategies during the third year in the current study showed that 1) as-needed injections based on quarterly examinations resulted in poor visual outcomes, 2) as-needed injections based on monthly examinations maintained the vision of good responders during the 2-year treatment with as-needed injections based on monthly examinations, 3) monthly injections maintained the vision of poor responders during the 2-year treatment with as-needed injections based on monthly examinations, and 4) the treat-and-extend schedule significantly improved the vision of the moderate responders during the 2-year treatment with as-needed injections based on monthly examinations. Recent studies [3,5-7,22,23] have found that strict treatment schedules are needed to obtain the optimal effect of ranibizumab treatment over the long term. Proactive treatment strategies such as monthly injections and a treat-and-extend schedule, which require frequent reinjections compared to as-needed injections based on monthly examinations, may be considered in eyes in which the outcomes of as-needed injections based on monthly examinations were unfavorable.

The goal of this study was that patients continue to be treated and follow the treatment schedule and that optimal visual outcomes are maintained. Since only nine (7%) of 137 eyes had discontinuous follow-up examinations during the third year, the treatment strategy in this study could avoid losing patients to follow-up and continue treatment. Previously published data [24] from the MARINA [2] and ANCHOR [1] trials showed that the 25-item National Eye Institute Visual Function Questionnaire scores improved whether patients were treated in the better or worse eyes, but there was difficulty reaching a clinically meaningful range [25-27] in the worse-seeing eyes compared to the better-seeing eyes. Maintaining treatment compliance and avoiding interruptions in follow-up visits, consideration of the VA of the fellow eye should be necessary to select the treatment strategy. Furthermore, when dominant eyes are treated and the fellow eyes have more severe visual loss, even the temporary VA loss caused by the recurrent exudative changes should affect the ability of patients to perform their ADLs. Such patients want to avoid VA fluctuations and may reject the as-needed injection schedule. Thus, a monthly injection schedule was selected for those patients in the current study.

At the start of the third year, the disadvantage associated with quarterly examinations was anticipated and this concern was shared with the patients and families. When the treatment criteria with quarterly examinations were considered, it was believed that even if the VA decreased during the third year, the disruption of the ADLs would be minimal. The mean VA increased from baseline to month 12 in the quarterly dosing groups in the EXCITE study [28] by 4.0 letters of the Early Treatment of Diabetic Retinopathy Study (ETDRS) chart in the 0.3 mg of ranibizumab quarterly group and 2.8 letters in the 0.5-mg quarterly group, whereas in the PIER study [29] that compared the efficacy of quarterly dosing of ranibizumab with sham treatment, although superior to sham treatment, the VA decreased over the 12-month study period to −1.6 letters in the 0.3-mg quarterly and −0.2 letter in the 0.5-mg quarterly groups. In the current study, the baseline VA was not maintained in eyes treated with as-needed injections based on quarterly examinations. Those eyes responded poorly to as-needed injections based on monthly examinations during 2 years.

In the current study, three (2%) of 128 eyes had minute amounts of vitreous hemorrhage after the injection during the 3-year follow-up. Since no new hemorrhage caused by PCV lesions was observed, the vitreous hemorrhage was suggested to be associated with the injection site in the uvea. In the 2-year results from the MARINA [1] and ANCHOR [2] studies, the incidence of vitreous hemorrhage was reported to be about 2%.

Since progression of retinal pigment epithelium (RPE) atrophy is common in the natural history of PCV [9,10] and photodynamic therapy (PDT) [30,31], progression of RPE damage during the long-term follow-up period of therapy in eyes with PCV may result in deteriorated visual function. Thus, in the current study, PDT was not considered as a treatment strategy for PCV during the third year.

A limitation of the current study was that about 21% of the eyes of prospective treatment-naïve Japanese patients who received ranibizumab therapy for 2 years could not participate in this study. This large number of excluded patients may have introduced bias into the outcomes, but this is a scenario in an ordinary clinic. Another limitation was that only ranibizumab was available to treat neovascular AMD in Japan during the current study. Recently, other anti-VEGF agents have been used to treat neovascular AMD. Switching from ranibizumab to another anti-VEGF agent may be an option for long-term follow-up [32]. Furthermore, the results of each treatment schedule during the third year were not simply compared, because the baseline VA levels and responses to ranibizumab during the 2-year treatment differed among the groups. Thus, the results of this study could not confirm which treatment schedule was most effective.

## Conclusions

Despite the limitations, the potential to prevent VA deterioration cannot be ignored, considering the current encouraging long-term treatment outcomes. The improved VA at the end of the second year compared to baseline can be maintained for the third year in patients with PCV treated with ranibizumab. PCV is a chronic disorder and the current findings indicated that the need for anti-VEGF treatment continues in patients with PCV. Since discontinuation of the follow-up will result in decreased VA, close follow-up should be warranted [14]. However, since the degree of patient satisfaction with the results of ranibizumab treatment and the status of the fellow eye vary, adherence to and motivation to continue ranibizumab treatment likely also vary among patients, and a uniform treatment strategy seems to be difficult to continue during long-term follow-up in all patients. Thus, individualized treatment strategies based on each patient’s situations should be necessary to not interrupt follow-up. The current study indicated that individualized treatment strategies based on each patient’s clinical course during 2 years maintained the improved VA during the third year. Additional studies are warranted to establish a reasonable treatment schedule for patients with PCV who must be followed for the remainder of their lives.
